# Developing a checklist to inform data linkage study designs for health technology assessments: a case study linking the Cardiac Rhythm Management (CRM) register to the Secure Anonymised Information Linkage (SAIL) Databank

**DOI:** 10.23889/ijpds.v1i1.314

**Published:** 2017-04-19

**Authors:** Ruth Louise Poole, Megan Dale, Antony Robert Wilkes, Hannah E Patrick, Mauro Lencioni, Michael Griffith, Chris P Gale, Grace Carolan-Rees

**Affiliations:** Cedar, Cardiff University; Cedar, Cardiff & Vale University Health Board; Independent Consultant (Honorary Statistical Advisor); National Institute for Health and Care Excellence; University Hospitals Birmingham NHS Foundation Trust; National Institute for Cardiovascular Outcomes Research

## Objectives

Health services researchers are increasingly engaging with the emerging field of data science, but relatively few have the expertise to understand how innovative data linkage methodologies can, and cannot, be successfully applied in practice. There is little published guidance written specifically for this purpose.

We aimed to develop study design criteria to help researchers in considering whether these methods are appropriate for their projects. A secondary objective was to test the criteria in a case study, and evaluate the application of the data linkage approach.

## Approach

Clinical procedures requiring further research (according to the National Institute for Health and Care Excellence) were assessed against newly-developed CINDER criteria (Coverage; Identifiers; Numbers; Data; Existing records; Retrieval) to check the suitability of using data linkage methods. The CALON (Cardiac Ablation: Linking Outcomes for NICE) study was then established to evaluate outcomes of cardiac ablation procedures.

Records from the UK’s Cardiac Rhythm Management (CRM) register were linked to routinely-recorded primary care, secondary care and mortality data in the Secure Anonymised Information Linkage (SAIL) Databank. Demographic profiles of patients identified from the register were compared with a group identified from SAIL. Outpatient attendances before and after ablation were compared using a Generalised Linear Mixed Model, assuming significance of p<0.05. Kaplan-Meier analyses were used to estimate survival.

Evaluation of methodological success concentrated on: adequately characterising patient populations from existing data; matching individuals between datasets; and data completeness/consistency.

## Results

The linked dataset contained 2220 anonymised records. Almost all (99.7%) patients from the register were matched using deterministic and probabilistic techniques. These patients were similar to individuals identified from hospital records with respect to sex and comorbidity score (p>0.05); mean age differed by 1.6 years (95% CI 0.23-3.04; p=0.02).

Patients accessed 26.7% fewer hospital outpatient appointments after ablation (95% CI 23.4 to 29.8; p<0.001). There was no significant difference in the number of primary care events before and after ablation (95% CI -4.3 to 4.0; p=0.91). Survival was estimated at 91.0% after 5 years. Insufficient granularity of data precluded subgroup analyses.

## Conclusions

Data linkage can be used to evaluate outcomes of interventions, although there are limitations associated with secondary use of observational data. The CALON study identified a post-ablation reduction in hospital attendances, suggesting an overall improvement in general health.

We aim to publish the full CINDER checklist as a generic resource to facilitate assessment of the feasibility of using data linkage methods in other projects.

